# Evolution of vortex-cavitation coherent structures in self-excited cavitation waterjets under sound waves excitation^☆^

**DOI:** 10.1016/j.ultsonch.2025.107634

**Published:** 2025-10-19

**Authors:** Zhenlong Fang, Houwen Yu, Bowen Hou, Shidong Fan, Xiangshu Lei, Xiaofeng Guo, Wenjiang Hou

**Affiliations:** a Sanya Science and Education Innovation Park of Wuhan University of Technology, Sanya 572025, China; b State Key Laboratory of Maritime Technology and Safety, Wuhan University of Technology, Wuhan 430063, China; c School of Transportation and Logistics Engineering, Wuhan University of Technology, Wuhan 430063, China; d Xiaomi Technology (Wuhan) Co., Wuhan 430078, China; e National Key Laboratory of Nuclear Reactor Technology, Nuclear Power Institute of China, Chengdu 610041, China; f LIED, UMR 8236, CNRS, Université Paris Cité Paris F-75006, France

**Keywords:** Self-excited cavitating waterjets, Ultrasonic excitation, Vortex-cavitation interaction, Proper orthogonal decomposition

## Abstract

Self-excited cavitation waterjets have been widely employed in surface treatment, material cutting, and equipment cleaning owing to their low cost and high energy conversion efficiency. This study investigates the evolution of vortex-cavitation coherent structures in self-excited cavitation waterjets under ultrasonic excitation using Large Eddy Simulation (LES) and Proper Orthogonal Decomposition (POD). External vibrational excitation was applied via a user-defined vibrating boundary within the Helmholtz nozzle chamber. The results showed that synchronization between vortex shedding and cavitation evolution was enhanced at an resonant frequency excitation of *f_e_* = 2.029 kHz. In contrast, long-distance cavitation bubble transport was enhanced, while dominant microbubble collapse fragmented vortex structures under ultrasonic excitation at *f_e_* = 25 kHz. POD analysis revealed that resonant excitation concentrated 80 % of the total energy in the first 200 modes, highlighting the dominance of vortex-induced cavitation. High-frequency excitation dispersed energy more broadly, with only 50 % of the total energy captured by the first 200 modes. Although the first vorticity mode remained large-scale, the second to fourth modes revealed disordered small-scale vortices due to intensified shear. These results elucidate the dynamic interplay between vortices and cavitation under ultrasonic excitation and provide a theoretical foundation for the active optimization of cavitation waterjet performance.

## 1 Introduction

Self-excited cavitation waterjets attracted considerable attention due to their powerful capabilities in energy exploration, rock fragmentation, and reservoir stimulation[1]. Their high energy conversion efficiency and low operating costs also make them widely applied in surface treatment, material cutting, and equipment cleaning[[2], [3], [4]]. The Kelvin-Helmholtz instability amplifies shear-layer disturbances and promotes upstream vortex shedding, resulting in the formation of large-scale vortex rings. This process enables periodic energy feedback that induces pressure oscillations and induces cavitation[[5], [6], [7]]. The induced cavitation and frequency oscillations significantly enhance the impact force of the self-excited cavitation waterjets.

Liu et al.[8] developed a modified theoretical model demonstrating that cavitation clouds dominate the oscillation frequency under low-pressure conditions. Fang et al.[9] utilized Large Eddy Simulation to capture the coupled evolution of vortices and cavitation. Fan et al.[10] experimentally investigated the expansion of vortex rings using high-speed photography. Similarly, Liu et al.[11] simulated the formation and propagation of stress waves during rock breakage, and Chen et al.[12] applied these jets in deep-sea mining, correlating rock porosity with breaking performance. Furthermore, Huang et al.[13] experimentally revealed that an optimal chamber length maximizes rock mass loss. Collectively, these studies underscore the significance of flow-structure interactions; however, the precise role of coherent vortex-cavitation structures remains insufficiently understood.

The mechanisms of self-excited oscillation are generally categorized into active and passive excitation[14]. Passive excitation, favored for its structural simplicity, has been extensively investigated through nozzle parameter optimization. For instance, Li et al.[15] experimentally examined the effects of nozzle inner-surface roughness, while explored the influence of nozzle lip geometry on the Strouhal number and erosion characteristics[16,17]. Shi et al.[18] found that the exit aspect ratio significantly influences pressure oscillations. Other researchers have analyzed the periodic dynamics induced by operating parameters[19] or designed novel aspiration methods to increase impact force[20]. Zhang et al.[21] further established a resonance-based correlation between oscillation frequency and chamber length. Despite these advancements, passive excitation often proves insufficient under complex operating conditions, motivating the exploration of active excitation strategies.

Vortex-cavitation interactions play a critical role in determining the dynamic behavior of these jets. Wang et al.[22] employed the Liutex-Omega method to analyze the evolution of vortex structures. Fang et al. [23] compared the coherent structures of jets produced by different nozzle types. In confined systems, Zdanowski et al. [24] found that nozzle configuration strongly affects flow asymmetry and vortex dynamics. Wu et al. [25] experimentally investigated the cavitation behavior of Helmholtz-type self-excited oscillating jets using high-speed photography, thereby validating numerical simulations of internal flow. Hou et al. [26] studied vortex-wall interactions, linking them to cavitation cloud expansion. These studies demonstrate the high sensitivity of vortex-cavitation coupling to boundary conditions, yet the quantitative pathways of energy transfer remain poorly characterized.

Proper Orthogonal Decomposition (POD) has emerged as a powerful method for investigating cavitation dynamics. Wang et al. [27] and Peng et al. [28] applied POD to confirm the periodicity of cavitation cloud shedding. Xu et al. [29] developed POD to propose predictive models for cavity length, and Hu et al. [30] experimentally related cavitation intensity to chamber geometry. More advanced studies have combined LES with decomposition methods, such as Dynamic Mode Decomposition (DMD), to reveal how inlet pressure and nozzle type determine modal energy distributions[31,32]. Ge et al. [33] further distinguished three regimes of cavitation instability, applying both DMD and POD to identify their coherent structures. Although these studies successfully capture key aspects of cavitation dynamics, they provide limited insights into the underlying vortex structures. Consequently, a need persists decouple vortex and cavitation modes to systematically analyze their coupled evolution.

Most prior research has emphasized cavitation cavitation erosion performance and erosion morphology characterization. Comparatively fewer efforts have been directed towards enhancing waterjet performance through active regulation. Related research on vortex dynamics has investigated topics such as the effect of inflow uniformity on tip-leakage vortices[34] and the influence of cavitation on Kelvin-Helmholtz instability growth[35]. Others have proposed new correlations for cavitation inception that incorporate structural interaction[36]. While applied studies have identified optimal parameters for marine fouling cleaning[37], proposed novel pulsed jet structures[38], and identified typical erosion patterns under high pressure[39], the absence of effective and versatile active-control strategies continues to limit the full potential of self-excited cavitation waterjets.

In this study, we address this research gap by investigating the spatial and temporal evolution of vortex-cavitation coherent structures in self-excited cavitation waterjets under ultrasonic excitation. By applying a vibrating boundary condition within the chamber of a Helmholtz nozzle, we establish an acoustic-flow coupling framework that enables active modulation of vortex-cavitation interactions. This approach aims to elucidate the mechanisms governing energy transfer between vortices and cavitation structure and to provide a theoretical foundation for the active optimization of cavitation waterjet performance.

## 2 Numerical approach

### 2.1 Geometric configuration and boundary conditions

**Fig. 1:**
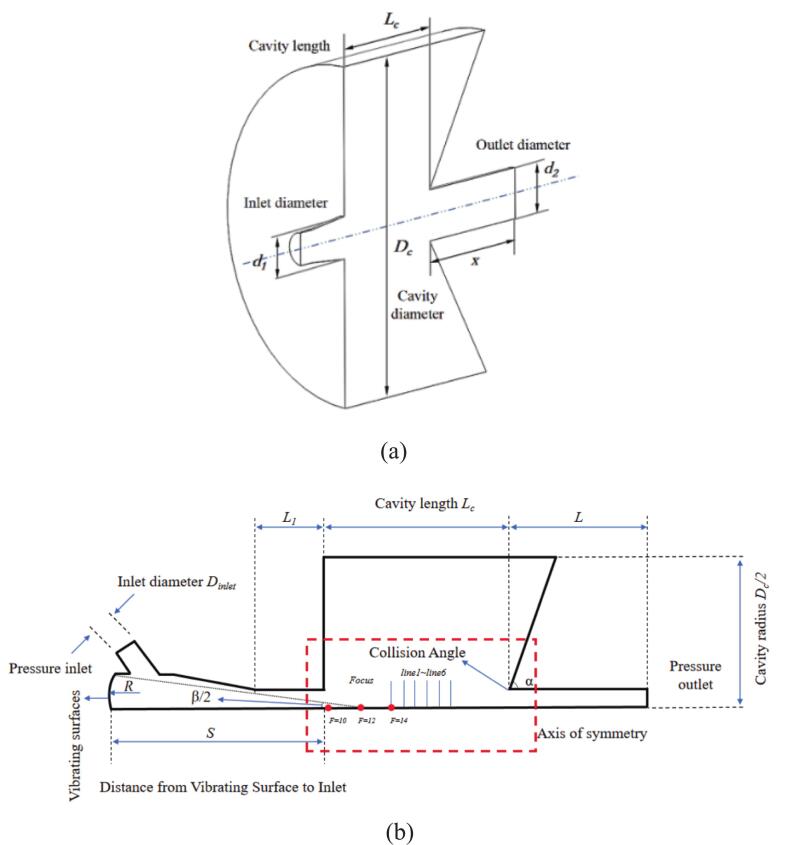
(a) Helmholtz nozzle, (b) excitation structure parameter design.

**Fig. 2:**
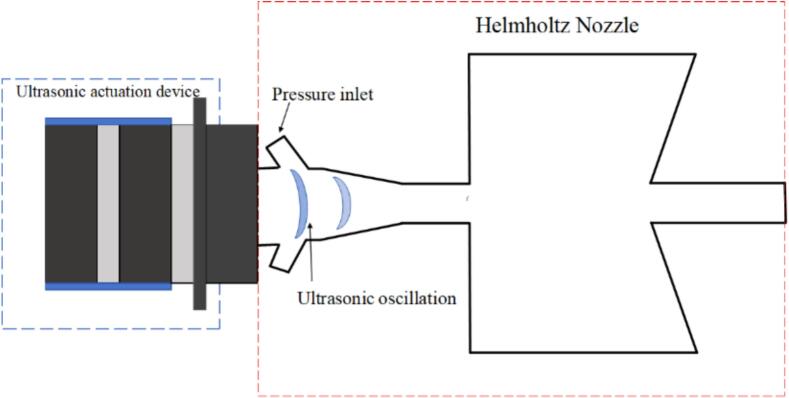
Schematic of the ultrasonic actuation device.

The geometry of Helmholtz nozzle is illustrated in Fig. 1(a), with geometric parameters adopted from Fang et al[9]. The ultrasonic actuation mechanism is illustrated in Fig. 2. In the simulation, ultrasonic actuation was represented by vibrating surfaces, with the vibrating surface corresponding to the left side of the nozzle chamber. As shown in Fig. 1(b), the ultrasonic excitation is applied via a user-defined vibrating boundary denoted as Vibration surfaces located at the left of the Helmholtz nozzle. Fluid entered the chamber through symmetrically inclined channels on both sides and exited after being excited by ultrasonic excitation within the chamber.

When the upstream excitation structure was applied, the corresponding parameters are listed in Table 1. An arc-shaped vibration surface representing the ultrasonic transducer was placed on the left side of the chamber, with its center aligned along the central axis of the waterjets.

**Table 1:** Helmholtz nozzle and excitation structure parameters.

*d_1_（*mm*）*	*d_2_*/*d_1_*	*D_c_*/*d_1_*	*L_c_*/*d_1_*	*α*	*D_inlet_*/*d_1_*	*x*/*d_1_*	*S*/*d_1_*	*β*	*R*/*d_1_*
2.6	1.2	8	3	60°	1	3.85	3.85	11.77°	0.96

**Fig. 3:**
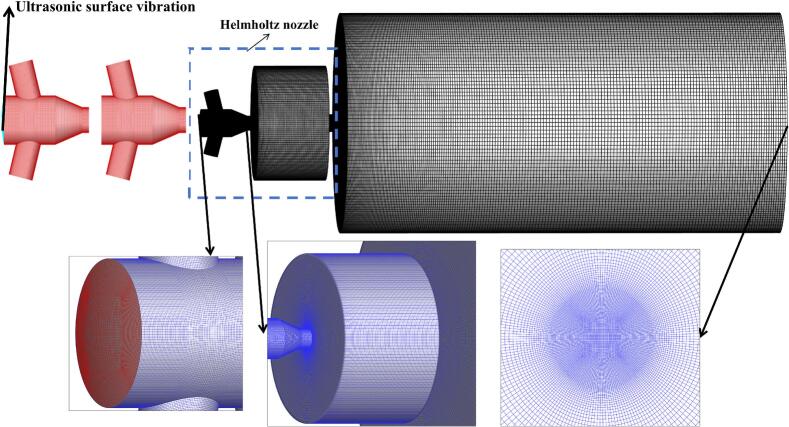
Schematic diagram of the computational model and grid. The blue frame indicates the location of the excitation structure parameter design shown in Fig. 1 (b).

Fig. 3 illustrates the computational grid configuration and local mesh refinement. The near-wall *y +* value was approximately 1, satisfying the grid resolution requirements for LES.

Grid independence was assessed based on the mean velocity at the nozzle outlet. As the nozzle grids increased from 5.5 million to 6.5 million, the monitored velocity exhibited negligible variation. Considering both computational accuracy and efficiency, a mesh with 6.0 million grids was adopted in nozzle region. An additional 3.5 million grids were assigned to the external flow field, resulting in a total of 9.5 million grids across the entire three-dimensional computational domain.

### 2.2 Numerical schemes and boundary conditions

The operating condition was set to P = 101.325  kPa. Water was selected as the primary phase, with a saturated vapor pressure of 3540 Pa. Bilateral inlets and the outlet were defined as pressure boundaries. No-slip conditions were applied to the impact wall, sidewalls, and pipe surfaces. The WALE subgrid-scale model was employed with a constant value of 0.325, and the complete numerical schemes are summarized in Table 2.

**Table 2:** Solution formulation.

Solver Settings	Format
Sub-lattice (SGS) model	WALE
Speed-pressure coupling solution	Coupled
Multiphase flow	Mixture
Cavitation model	Schnerr-Sauer
Spatial discretization scheme	Bounded Central Differencing
Time discrete format	Second Order Implicit
Calculate the simulation step sizeΔt	5 × 10^-6^ s

It should be noted that in the present simulations, the Schnerr-Sauer cavitation model was employed within an LES framework. The attenuation of ultrasonic waves by cavitation bubbles during the simulation process indeed needs to be considered, as the sound waves emitted by adjacent bubbles can significantly affect bubble pulsation behavior. The duration and magnitude of the ultrasonic pressure at each instant exert a distinct influence on the variations of bubble radius, temperature, internal pressure, and mass content [40].

Experimental studies have demonstrated that ultrasonic excitation can markedly enhance jet performance[1]. Using high-speed imaging and particle image velocimetry (PIV), researchers have captured acoustic cavitation patterns and identified two distinct modes of bubble motion on the radiating surface: (1) the formation, aggregation, and coalescence of cavitation bubbles, and (2) the aggregation, shrinkage, expansion, and collapse of bubble clusters[41]. Under small pressure amplitudes, cavitation bubbles may fragment into smaller ones, with the Laplace pressure gradient acting as the final trigger for fragmentation[42]. Computational fluid dynamics (CFD) and dynamic mesh techniques have been shown to effectively simulate the cavitation characteristics of ultrasonic cavitation waterjets[43,44]. However, these studies have not explicitly addressed the attenuation of ultrasonic waves caused by cavitation bubbles.

The main objective of this study is to capture the quasi-periodic evolution of vortex-cavitation coherent structures and to perform modal decomposition analysis, rather than resolving detailed acoustic coupling effects. For similar purposes, many recent studies have adopted simplified cavitation models that neglect bubble–bubble acoustic coupling while still achieving good agreement with experimental observations of cavitating jets [3,5,9].

Recent investigations have further indicated that bubble–bubble interactions may influence acoustic cavitation in dense bubble clusters [45] or through delayed acoustic wave coupling [46], and that thermoacoustic effects can become significant in focused ultrasound fields [47]. Nevertheless, these complex mechanisms remain beyond the scope of the present work. Future research will incorporate advanced multi-bubble interaction models, such as coupled Rayleigh-Plesset-type formulations or cluster interaction frameworks, to explicitly account for ultrasonic attenuation and bubble–bubble acoustic coupling.

### 2.3 Cavitation model

To capture the transient cavitation phenomena in the jet flow, the Schnerr-Sauer cavitation model was employed. This model is widely adopted owing to its computational robustness, rapid convergence, and capability to efficiently simulate cavitation bubble dynamics without introducing additional empirical constants.

The transport of the liquid and vapor phases is governed by the following continuity equations:

Continuity equation for the liquid phase:

(1) $$\frac{\partial }{\partial t}\left[\left(1-\alpha \right)\rho_{l}\right]+\nabla \cdot \left[\left(1-\alpha \right)\rho_{l}\vec{V}\right]=-R$$

Continuity equation for the vapor phase:

(2) $$\frac{\partial }{\partial t}\left(\alpha \rho_{v}\right)+\nabla \cdot \left(\alpha \rho_{v}\vec{V}\right)=R$$

Mixture:

(3) $$\frac{\partial }{\partial t}\left(\rho \right)+\nabla \cdot \left(\rho \vec{V}\right)=0$$

Where *l* corresponds to the liquid term and *v* corresponds to the gas term.

The mixture density *ρ* is defined as:

(4) $$\rho =\alpha \rho_{v}+\left(1-\alpha \right)\rho_{l}$$

The relationship between mixing density and vapor volume fraction (*α*):

(5) $$\frac{D\rho }{\mathrm{Dt}}=-\left(\rho_{l}-\rho_{v}\right)\frac{D\alpha }{\mathrm{Dt}}$$

The correlation between vapor volume fraction α and bubble number $n_{b}$ and bubble radius in unit liquid volume $R_{B}$:

(6) $$\alpha =\frac{n_{b}\frac{4}{3}\pi R_{B}^{3}}{1+n_{b}\frac{4}{3}\pi R_{B}^{3}}$$

(7) $$R_{B}={\left(\frac{\alpha }{1-\alpha }\frac{3}{4\pi }\frac{1}{n}\right)}^{\frac{1}{3}}$$

Net phase transition rate (R):

(8) $$R=\frac{\rho_{\nu }\rho_{l}}{\rho }\alpha \left(1-\alpha \right)\frac{3}{R_{B}}\sqrt{\frac{2}{3}\frac{\left(P_{\nu }-P\right)}{\rho_{l}}}$$

The Schnerr-Sauer cavitation model was employed, in which cavitation nuclei were assumed to be uniformly distributed with a single initial radius of $R_{B}$ = 1 × 10^-6^m and a constant bubble number density of $n_{b}$ = 1 × 10^13^m^−3^. The same parameters were applied for cases with and without ultrasonic excitation to ensure consistency.

### 2.4 Model validation

The numerical model was previously validated by Fang et al.[23], by comparing the simulated cavitation evolution with high-speed snapshots. As summarized in Table 3, the simulated axial pressure oscillations exhibited a maximum deviation of 10.63 % and an average deviation of 9.26 % relative to experimental measurements, demonstrating acceptable agreement.

**Table 3:** Comparison of experimental and simulated axial pressure in the nozzle.

Inlet pressure（MPa）	*d_1_（*mm*）*	*d_2_*/*d_1_*	*D_s_*/*d_1_*	*D*/*d_1_*	*L*/*d_1_*	Axial pressure（MPa）		Error
						Experiment	CFD	
10	2	1.1	3	12	2	19.21	20.67	7.60 %
15					3	15.13	16.64	9.98 %
20		1.2	6.5		2	24.92	27.57	10.63 %
25					1.5	31.81	34.62	8.83 %

## 3 Results and discussion

In this study, the numerical simulations based on the LES were performed using ANSYS Fluent to resolve the unsteady turbulent structures within the nozzle and outflow field. The POD was subsequently carried out in MATLAB to extract dominant coherent structures and quantify their spatial and temporal characteristics.

**Fig. 4:**
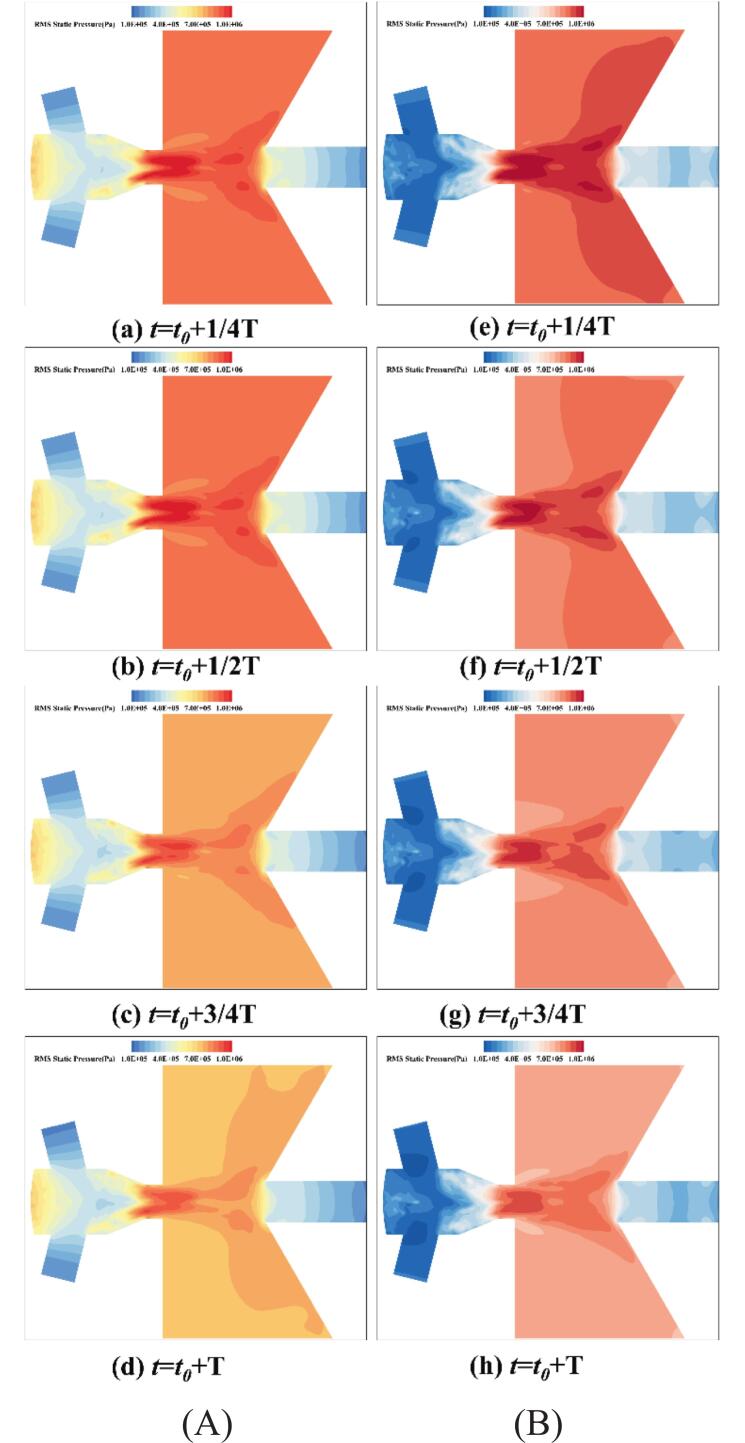
Pressure fluctuations of self-excited waterjets within the Helmholtz chamber. (A) *f_e_* = 25 kHz. (B) *f_e_* = 0 kHz. (Ultrasonic excitation amplitude A = 10 μm).

Fig. 4(a-d) show that under ultrasonic excitation, the vibration of the transducer induces a pronounced oscillating pressure. The waterjets with periodic pressure fluctuations enters the Helmholtz oscillator and undergoes further resonance, thereby enhancing the cavitation and oscillation characteristics of the waterjets. where the waterjet, modulated only by the Helmholtz oscillator, enters the external flow field. Under ultrasonic excitation, the flow exhibits distinctly periodic pressure fluctuations.

### 3.1 Evolution of vortex structures under ultrasonic excitation

**Fig. 5:**
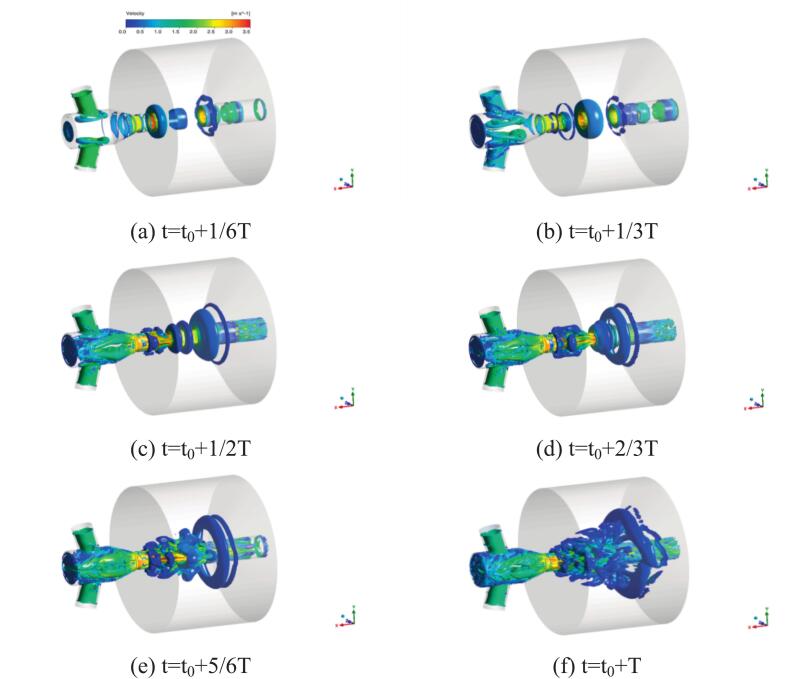
Evolution process of vortex structures inside the chamber under 25 kHz ultrasonic excitation. (Ultrasonic excitation amplitude A = 10 μm).

Fig. 5 illustrates the evolution of vortex structures within the chamber under ultrasonic excitation. In Fig.3(a)-Fig.3(b), ultrasonic excitation intensified shear, amplifying shear-layer disturbances.

The migrating vortex rings formed large-scale vortices, which eventually collided and fragmented near the downstream impact wall. In Fig. 5 (c)-Fig. 5(d), pressure fluctuations induced flow instability near the shear layer, accompanied by increased shear strength. Small-scale vortices formed along the wall and interacted with the coherent structures. These pressure waves propagated upstream, resulting in vortex shedding in Fig. 5 (f), the vortex expanded along the impact wall before breaking down, while the shear layer gradually stabilized.

#### 3.1.1 Influence of excitation frequency on flow field resonance

**Fig. 6:**
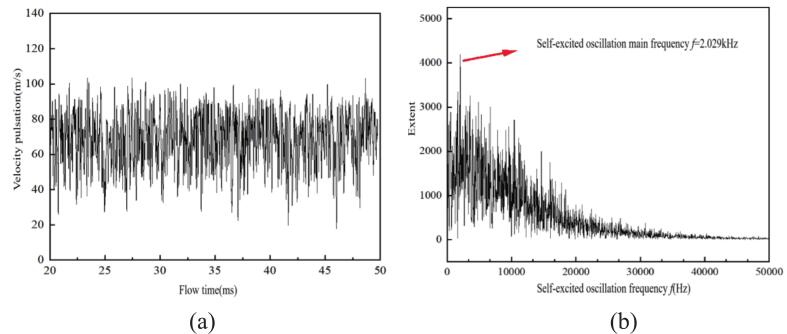
The velocity pulsation and spectrum of self-excited cavitation waterjets (*f_a_* = 2.029 k Hz, P _inlet_ = 5.0 MPa). (a) Velocity pulsation. (b) velocity spectrum. (Ultrasonic excitation amplitude A = 10 μm).

As illustrated in Fig. 6, resonance was observed when the external excitation frequency was *f_a_* = 2.029 kHz. It should be noted that this frequency falls within the audible sound range; however, it was selected as a representative case to investigate the flow field resonance phenomenon and for comparative analysis under various excitation conditions.

**Fig. 7:**
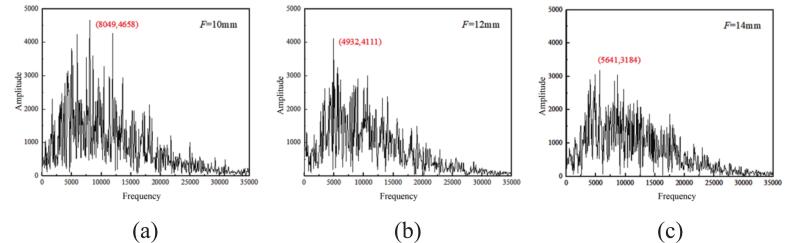
Time-frequency domain curves at different focal distances. (a) F = 10 mm, (b) F = 12 mm, (c) F = 14 mm (Ultrasonic excitation amplitude A = 10 μm).

As shown in Fig. 1(b), the focal distance of the active excitation in the simulation is adjusted by modifying the position and amplitude of the vibrating surface, thereby regulating the energy concentration of the ultrasonic actuation. Fig. 7 illustrates the dynamic response relationship between different focal distances and the jet oscillation characteristics. The oscillation frequencies and amplitudes corresponding to the FFT spectral peaks within the computational period are marked in the figure. When F = 10 mm, the acoustic field exhibits the strongest focusing effect, with energy concentrated near the nozzle throat. Cavitation occurs within a confined region, accompanied by the repeated generation and collapse of nascent bubbles. The imposed vibration modulates the jet stability, resulting in pronounced fluctuations in pressure amplitude. Therefore, at shorter focal distances, the concentrated acoustic energy induces a high-frequency and low-amplitude oscillation behavior.

When 12 mm ≤ F ≤ 14 mm, the oscillation frequency exhibits a negative correlation with the Strouhal number (*S_v_*), indicating that within this focal distance range, the enhancement of the shear layer is accompanied by a reduction in overall jet oscillation intensity. This trend suggests that when the focal distance deviates from the intermediate range of 12 mm ≤ F ≤ 14 mm, the acoustic-flow coupling induced by external vibration excitation enhances the frequency-domain response of the jet. However, within this range, the strengthening of the shear layer corresponds to a decline in oscillation intensity, while the two-phase mixing capability of the jet improves. Consequently, ultrasonic excitation enables dynamic regulation of the gas-phase distribution by modulating both the oscillation characteristics and the shear strength.

**Fig. 8:**
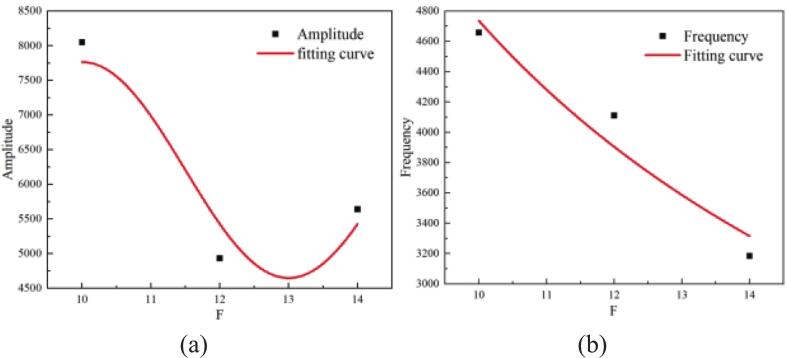
Fitted curves of amplitude and frequency variation with focal distance. (a) Amplitude; (b) Frequency.

As shown in Fig. 8, the fitted curves of oscillation amplitude and frequency under different focal distances yield the following empirical relationships:

Amplitude:

(9) y=6207.3+1558.5×sin((F-2.5)π/3)

Frequency:

(10) $$y=54308.5\times F^{-1.059}$$

#### 3.1.2 Vortex evolution in outflow field

**Fig. 9:**
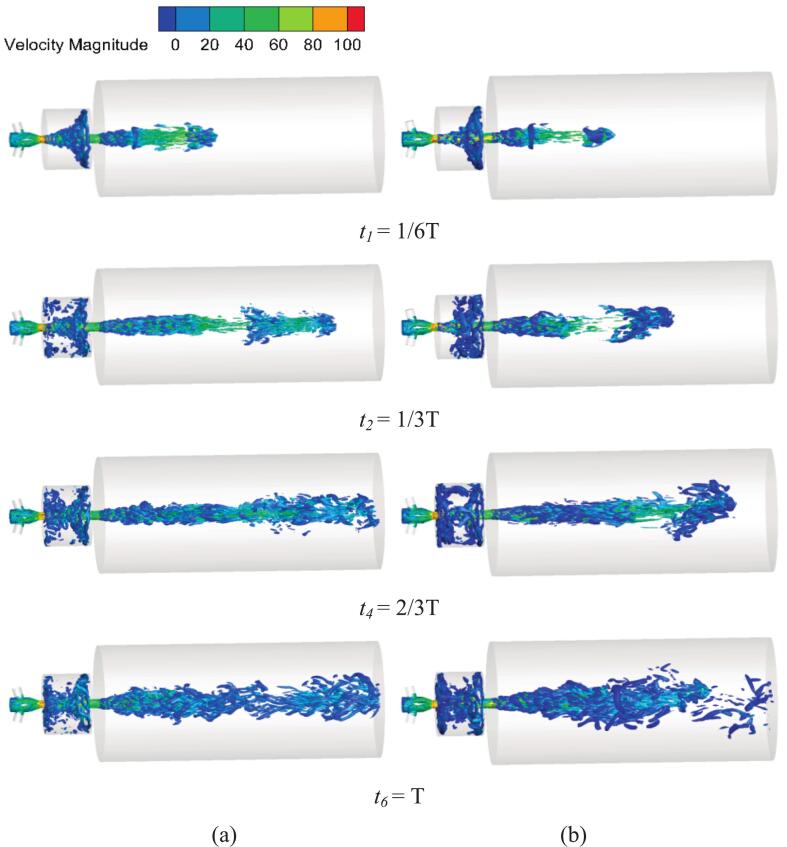
Vortex formation under different excitation conditions. (a) No vibration excitation (*f_e_* = 0 Hz). (b) resonance excitation (*f_e_ =* 2.029 kHz) (Ultrasonic excitation amplitude A = 10 μm).

Without vibrational excitation, the upstream nozzle generates fewer vortex rings of lower intensity. The primary vortex ring exhibits minimal deformation, and boundary-layer separation occurs only near the chamber impact wall. Separation along the wall boundary layer is not pronounced, with the vortices confined within a 20 angle from the waterjet axis. After the coherent structures interact with the impact wall, large-scale vortex breakdown occurs, followed by a slow dissipation process and low-frequency shedding of separated vortices, as shown in Fig. 9 (a). In Fig. 9 (b), under resonant excitation, the primary vortex stretches rapidly, and wall-boundary layer separation occurs earlier and more intensely, accelerating vortex shedding. The resonant excitation further promotes vortex breakup and amplifies pressure fluctuations.

#### 3.1.3 Analysis of spatial morphological differences of vortex structures under different excitations

**Fig. 10:**
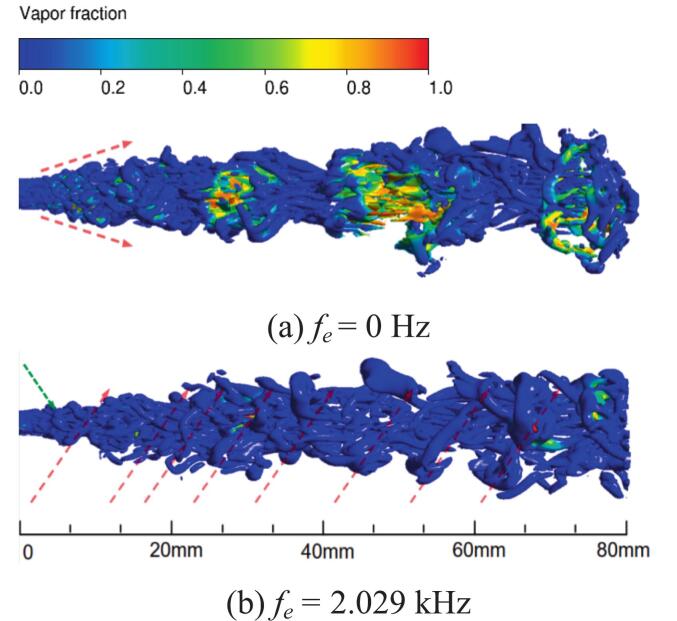
The fully developed waterjets vortex structures in different excitations. (Ultrasonic excitation amplitude A = 10 μm).

Fig. 10 reveals differences in waterjets vortex structures under different excitations. As shown in Fig. 10 (a), vortices were confined near the nozzle outlet with a short streamwise extent, and no large-scale structures developed because diffusion near the nozzle outlet was suppressed. Fig. 10 (b) illustrates that vortices were more extensively distributed along the waterjets axis and exhibited a complex, multi-scale interlaced pattern. The vortex array was characterized by closely spaced, well-organized coherent structures.

### 3.2 Analysis of time-averaged vortex characteristics

One of the basic structures of turbulence is eddies at various scales, including a background flow field consisting of many small ran dom eddies and large-scale eddy structures. The vortex intensity is a state function of spatial coordinates, independent of fluid micro clusters, and its components can be mathematically expressed as:

(11) $$\omega_{x}=\frac{\partial V_{z}}{\partial y}-\frac{\partial V_{y}}{\partial z}$$

(12) $$\omega_{y}=\frac{\partial V_{x}}{\partial z}-\frac{\partial V_{z}}{\partial x}$$

(13) $$\omega_{z}=\frac{\partial V_{y}}{\partial x}-\frac{\partial V_{x}}{\partial y}$$

Among the vortex structures of fluid motion, streamwise and spanwise vortices are two important features that represent the change in vorticity in the parallel and perpendicular directions to the flow, respectively. When comparing the characteristic sections of flow fields based on the vortex decomposition theory, the collapse time of vortex shedding is taken as the representative time. To facilitate an analysis of the vortex structure of the flow field, the streamwise vortices and spanwise vortices are made dimensionless and defined as follows.

Streamwise vortex

(14) $$\Omega_{n}=\frac{D}{V_{0}}\sqrt{\omega_{y}^{2}+\omega_{z}^{2}}$$

Spanwise vortex

(15) $$\Omega_{s}=\frac{D}{V_{0}}\omega_{x}$$

_Among them, D is the characteristic diameter, V0 is the characteristic velocity, and the jet is the average velocity of the outlet._

#### 3.2.1 Spanwise vortex evolution under various excitations

**Fig. 11:**
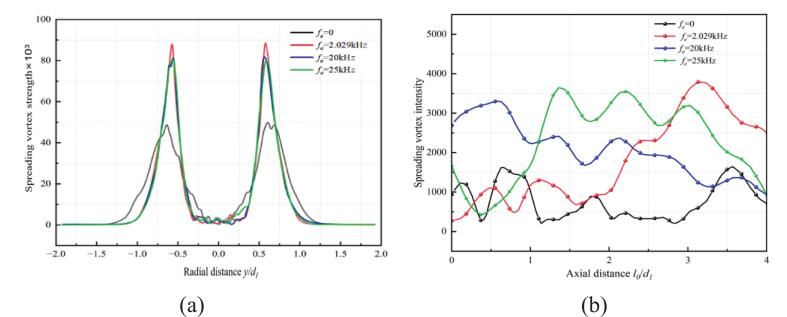
The spanwise vorticity magnitude at different excitation frequencies. (a) The radial distribution of vorticity magnitude. (b) Axial distribution of vorticity magnitude. (Ultrasonic excitation amplitude A = 10 μm).

As shown in Fig. 11 (a), peak vorticity consistently occurred bilaterally within the shear layer and decayed radially outward within 2*d_1_* downstream of the nozzle outlet. Under resonance-frequency excitation, the peak vorticity reached 8.9 × 10^4^ s^−1^, approximately 30 % higher than that observed under non-resonant frequencies. The non-resonant case produced in minimal vorticity generation. Fig. 11 (b) shows that within the *l_0_*/*d_1_* ≤ 1 region, 20 kHz and 25 kHz excitations induced vorticity distribution trends in the spanwise direction compared to those observed at lower frequencies or under non-resonant conditions.

#### 3.2.2 Characteristics of streamwise vortex structures

**Fig. 12:**
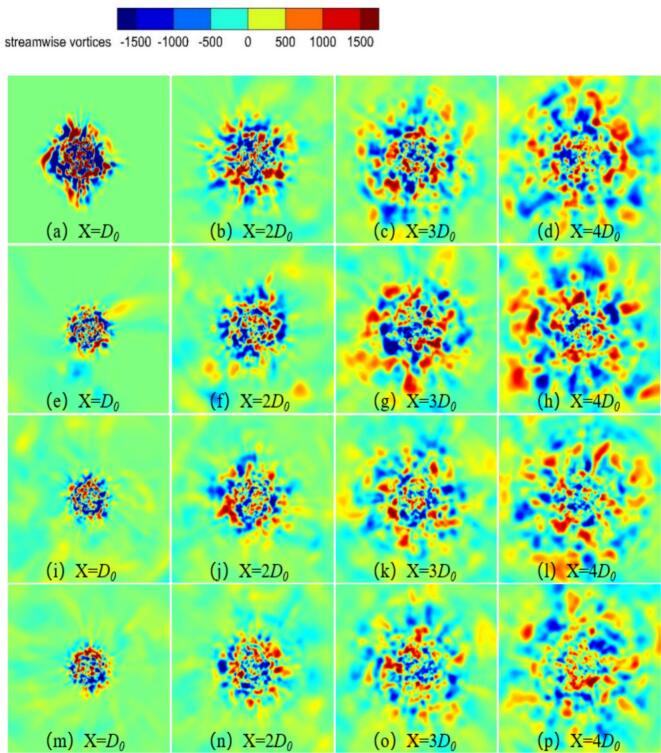
Comparison of time-averaged streamwise vortices in the Y-Z plane of of self-excited cavitation waterjets at different excitations. (a)-(d) *f_e_* = 0 Hz. (e)-(h) *f_e_* = 2.029 kHz. (i)-(l) *f_e_* = 20 kHz. (m)-(p) *f_e_* = 25 kHz. (Ultrasonic excitation amplitude A = 10 μm).

Fig. 12 illustrates the time-averaged distribution of streamwise vortices on the y-z plane at different downstream locations in the external flow field. As the excitation frequency increased, the streamwise vortices gradually contracted across the measured cross-sections. Vortex generation intensified significantly under resonance conditions at *f_e_* = 2.029 kHz. As the waterjets propagated downstream, the streamwise vortices exhibited increasing radial convergence. These results indicate that higher excitation frequencies promote contraction of the waterjet and flow energy. Consequently, boundary-layer stability was enhanced, accompanied by intensified vortex shedding at the nozzle outlet.

### 3.3 Cavitation characteristics under different ultrasonic excitations

**Fig. 13:**
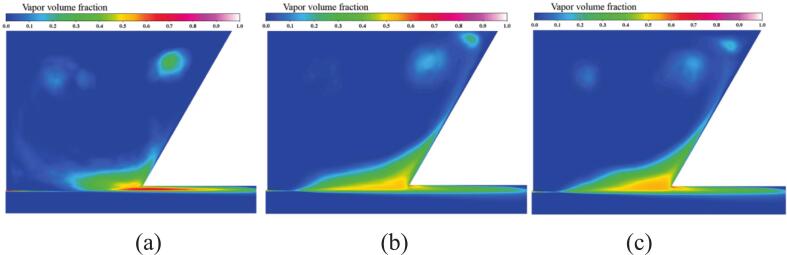
Time-averaged vapor volume fraction under different ultrasonic excitation frequencies. (a) *f_e_* = 0 Hz. (b) *f_e_* = 20 kHz. (c) *f_e_* = 25 kHz. (Ultrasonic excitation amplitude A = 10 μm).

Fig. 13 presents the time-averaged vapor distribution in the cavity with and without ultrasonic excitation. Under excitation, vapor accumulates on both sides of the shear layer with a larger area, indicating that excitation promotes cavitation over a wider range along the shear layer. High-frequency vibration enables continuous transmission of pressure waves into the waterjets, which couple with the jet-induced pulsating pressure generated within the oscillator and amplify the overall pressure fluctuations. The intensified pressure pulsation enhances cavitation near the shear layer at the main flow boundary, resulting in an increased number of vapor bubbles and their accumulation within the cavity.

In the absence of vibration excitation, the lack of sustained external energy input and the excessive flow velocity lead to the collapse of vortex structures, thereby weakening the feedback mechanism. Consequently, the velocity oscillations within the cavity are reduced, and due to the effect of fluid viscosity, the vapor circulation rate becomes slower, with part of the vapor remaining trapped inside the cavity, as shown on the left side of Fig. 13(a).

**Fig. 14:**
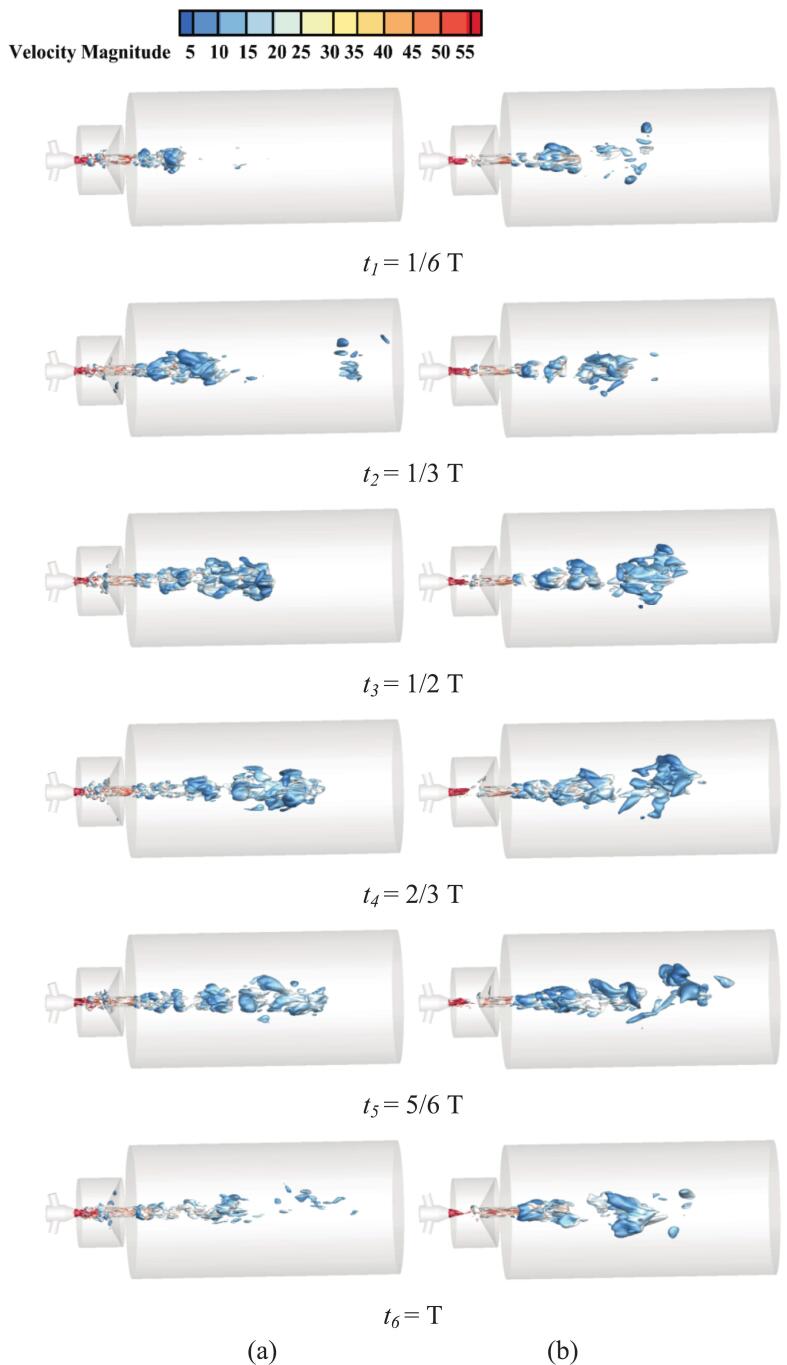
Evolution of the cavitation structure during the period under different under different ultrasonic excitation (P _inlet_ = 5 MPa, Q = 1 × 10^8^). (a) *f_e_* = 0 Hz. (b) *f_e_* = 2.029 KHz. (Ultrasonic excitation amplitude A = 10 μm).

As shown in Fig. 14, under resonant excitation, the dynamic mesh efficiently transfers the vibration energy into the nozzle oscillator, significantly enhancing the generation frequency and intensity of upstream vortex rings. The primary vortex is rapidly stretched, leading to a sharp increase in the local strain rate and an early. The combined effects of wall shear and vortex compression induce unstable three-dimensional vortex pairs. The cavitation expansion further elongates small-scale vortices, accelerating their detachment from the dominant frequency structures and intensifying vortex breakup and pressure oscillations. As shown in Fig. 14(b), the high self-sustainability of these vortical structures enables continuous cavitation inception throughout the excitation cycle, resulting in a wide cavitation region in the downstream jet field.

**Fig. 15:**
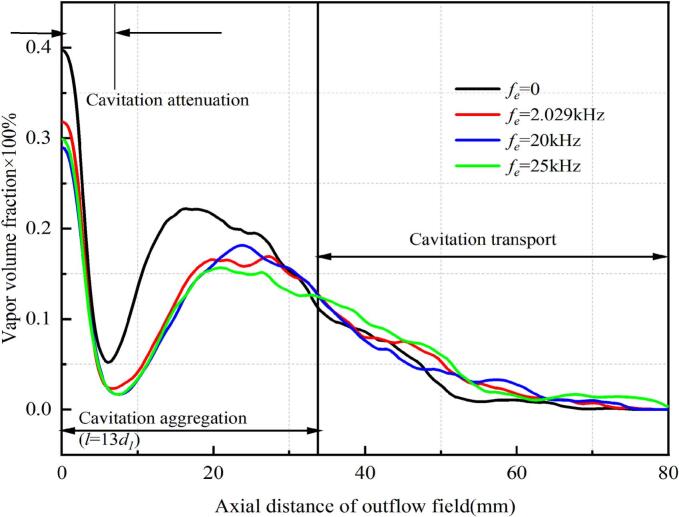
Comparison of vapor volume fraction distribution of external flow field axis under different ultrasonic excitations.

Fig. 15 illustrates the influence of different ultrasonic excitations on the time-averaged vapor volume fraction along the jet axis in the external field, revealing two distinct stages of cavitation distribution. In the region of *l_0_* < 13*d_1_*, the case without ultrasonic excitation exhibited a significantly higher vapor volume fraction than those with excitation. All cases showed a sharp initial decline followed by an inflection point within this region. In the region of *l_0_* ≥ 13*d_1_*, resonance and ultrasonic excitations produced higher vapor volume fractions. The 25 kHz excitation generated the highest vapor fraction by enhancing cavitation intensity. Moreover, the 25 kHz excitation promoted cavitation coalescence and long-range transport, thereby increasing cavitation erosion capability. The critical transition at *l_0_* = 13*d_1_* demarcates the boundary where near-field cavitation dominates in flows without ultrasonic excitation.

**Fig. 16:**
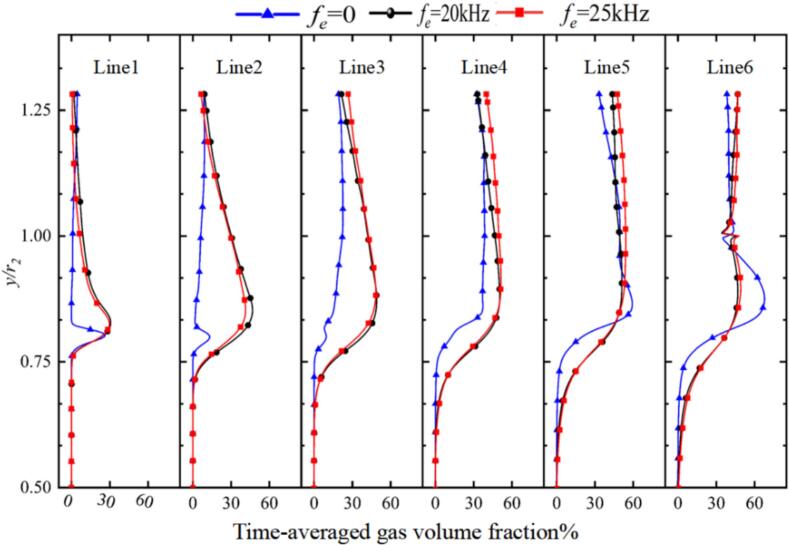
Time-averaged vapor volume fraction distribution along Lines 1 through 6 (Ultrasonic excitation amplitude A = 10 μm).

As shown in Fig. 1(b), the locations of the monitoring lines inside the chamber are illustrated. Fig. 16 presents the time-averaged vapor volume fraction at line 1–6 downstream of the nozzle exit. Under non-excitation conditions, the cavitation bubbles were confined to the near-wall region, and collapsed rapidly close to the nozzle exit. When ultrasonic excitation was applied at 20 kHz and 25 kHz, the transport distance of the bubbles was significantly extended to line 6. It is particularly noteworthy that the highest vapor volume fraction was observed at the 25 kHz frequency.

**Fig. 17:**
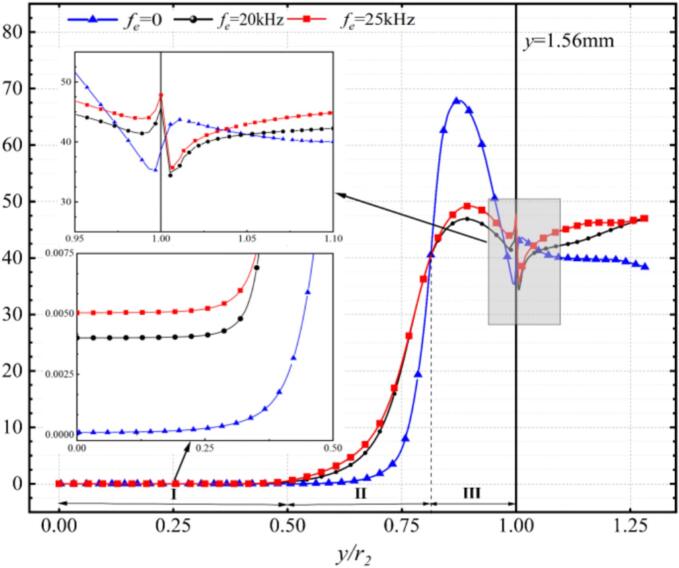
Time-averaged vapor distribution at cross-section Line 6, located at the chamber. (Ultrasonic excitation amplitude A = 10 μm).

Fig. 17 illustrates the time-averaged vapor distribution at cross-section Line 6, located at the chamber exit. The high-frequency ultrasonic excitation 25 kHz disrupts the coherence of large-scale vortices, generating dispersed microbubble clusters that propagate further before collapsing.

### 3.4 POD analysis of vortex-cavitation characteristics under varying excitation frequencies

#### 3.4.1 POD energy distribution

**Fig. 18:**
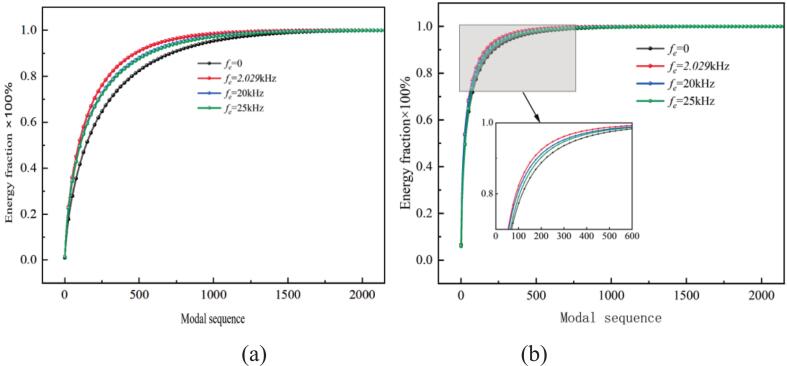
The energy accumulation of vortex and cavitation modes at different excitation frequencies. (a) Vortex mode energy accumulation. (b) Cavitation mode energy accumulation. (Ultrasonic excitation amplitude A = 10 μm).

Fig. 18 shows the cumulative energy of the first *i_th_* POD mode for the vorticity and cavitation fields under different excitation frequencies. The excitation frequency had a significant influence on the vortex and cavitation structures of the waterjets. The energy convergence rate at *f_e_* = 2.029 kHz was faster than at other frequencies.

#### 3.4.2 Comparative of vorticity and cavitation mode under different excitation frequency

**Fig. 19:**
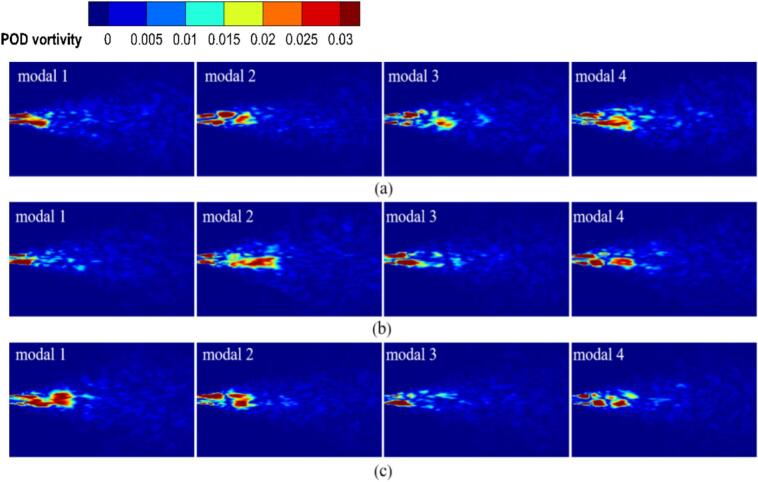
Comparison of vorticity modes under different excitation frequencies. (a) *f_e_ =* 2.029 kHz. (b) *f_e_ =* 20 kHz. (c) *f_e_ =* 25 kHz. (Ultrasonic excitation amplitude A = 10 μm).

**Fig. 20:**
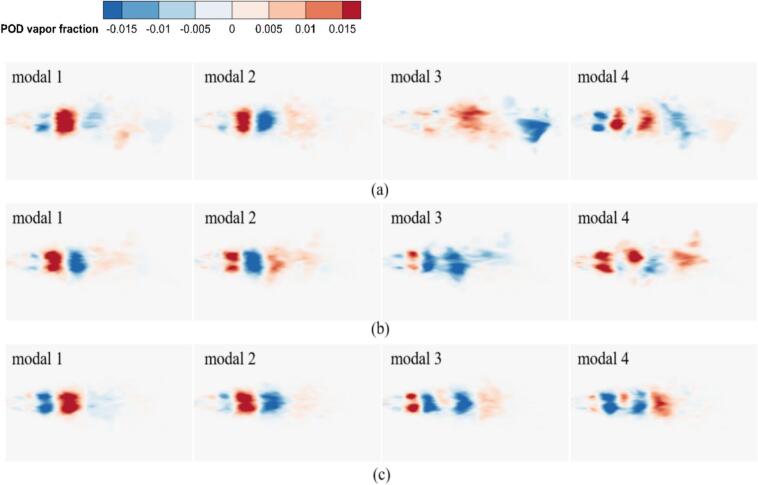
Comparison of cavitation modes under different excitation frequencies, (a) *f_e_ =* 2.029 kHz. (b) *f_e_ =* 20 kHz. (c) *f_e_ =* 25 kHz. (Ultrasonic excitation amplitude A = 10 μm).

In Fig. 19, The vorticity modes are defined as the spatial modes （φi(x)） extracted from the vorticity field data via POD. The modes are ordered by their energy content the corresponding eigenvalue $\lambda_{i}$, with the first mode representing the most energetically dominant spatial pattern of the vortex structures. The first four vorticity and cavitation mode under different excitation frequencies are shown in Fig. 19 and Fig. 20. At *f_e_* = 2.029 kHz, the first vorticity mode exhibited symmetric large-scale vortex structures. The corresponding cavitation mode in Fig. 20 (a) showed cavitation concentrated within the vortex cores, forming continuous band-like distributions. This indicates that primary cavitation is driven by vortex-induced low pressure. Fig. 18 confirms that both fields exhibited rapid energy convergence within the first two modes, with the first 200 modes capturing 80 % of total energy, reflecting strong periodicity.

In Fig. 20, the cavitation modes are defined as the spatial modes （φ_i_(x)） extracted from the vapor volume fraction field data via POD. The modes are ordered by their energy content the corresponding eigenvalue $\lambda_{i}$, with the first mode representing the most energetically dominant spatial pattern of the cavitation bubbles. At *f_e_* = 20.0 kHz, the first vorticity mode became asymmetric, while the second to fourth vorticity modes revealed irregular small-scale vortices. In the first cavitation mode, the high-energy region contracted, with bubble collapse occurring near the vortex cores. The second to fourth cavitation modes exhibited fragmented distributions and scattered energy. Ultrasonic excitation generated continuous microbubbles under intense shear.

At *f_e_* = 25 kHz, the first vorticity mode remained large-scale. However, the second to fourth vorticity modes exhibited disordered small-scale vortices, indicating that shear effects were enhanced by the high-frequency excitation. The first 200 modes captured 50 % of the total energy, resulting a broader dispersion of energy. This dispersion further reinforced the dominance of ultrasonic cavitation. High-frequency pressure waves drove rapid bubble motion, which disrupted secondary vortices and promoted cavitation under ultrasonic excitation.

## 4 Conclusion

This paper investigated the influence of different ultrasonic excitation frequencies on the evolution of vortex-cavitation coherent structures in self-excited cavitation waterjets. The main conclusions are as follows:

(1) External vibrational excitation significantly enhances the generation ability of shear vortices at the nozzle outlet. When the resonant frequency excitation was applied at *f_e_* = 2.029 kHz, the peak intensity of spanwise vortices increases by 30 %, and the periodic synchronization between large-scale vortices and cavitation is strengthened. In contrast, under ultrasonic excitation at *f_e_* = 25 kHz, long-distance cavitation bubble transport is facilitated. However, the correlation between vortex structures and cavitation weakens. Ultrasonic cavitation becomes dominant through the collapse of microbubbles, leading to vortex fragmentation and accelerated energy dissipation.

(2) High-frequency ultrasonic excitation enhances shear effects, giving rise to disordered small-scale vortices in the second to fourth vorticity modes and promoting broader dispersion of flow energy. The first 200 modes capture only 50 % of the total energy, reflecting a transition toward turbulence-dominated cavitation.

(3) Under resonant frequency excitation at *f_e_* = 2.029 kHz, the first 200 cavitation modes account for approximately 80 % of the total energy, indicating strong vortex-induced cavitation coupling. In contrast, high-frequency excitation at 20 kHz and 25 kHz leads to broader energy dispersion and enhances the dominance of ultrasonic cavitation.

While the present study elucidates vortex-cavitation interactions under isothermal conditions, the potential influence of thermodynamic effects merits further consideration. Incorporating non-isothermal cavitation models in future work will allow for a quantitative assessment of temperature fluctuations, providing deeper insight into cavitation dynamics and offering more accurate guidance for the design of cavitating devices operating under high-load or cryogenic conditions.

## CRediT authorship contribution statement

**Zhenlong Fang:** Writing – original draft, Funding acquisition, Conceptualization. **Houwen Yu:** Methodology, Investigation, Formal analysis. **Bowen Hou:** Validation, Resources, Data curation. **Shidong Fan:** Visualization, Software, Investigation. **Xiangshu Lei:** Supervision, Resources, Project administration. **Xiaofeng Guo:** Software, Project administration, Conceptualization. **Wenjiang Hou:** Writing – review & editing, Supervision, Project administration.

## Declaration of competing interest

The authors declare that there are no potentially competing financial interests or personal relationships that could influence the work reported herein.

## References

[1] Xiong J., Cai J., Kang Y., Wang X., Lai Q., Li D. (2024). Generation of effective pulsed waterjets by ultrasonic nozzle used for energy exploration. Energy.

[2] Li D., Kang Y., Ding X., Liu W. (2017). Experimental study on the effects of feeding pipe diameter on the cavitation erosion performance of self-resonating cavitating waterjet. Exp. Therm Fluid Sci..

[3] Liu W., Kang Y., Wang X., Liu Q., Fang Z. (2020). Integrated CFD-aided theoretical demonstration of cavitation modulation in self-sustained oscillating jets. Appl. Math. Modell..

[4] Xue Y., Si H., Hu Q. (2017). The propagation of stress waves in rock impacted by a pulsed water jet. Powder Technol..

[5] Ge M., Petkovšek M., Zhang G., Jacobs D., Delgosha O.C. (2021). Cavitation dynamics and thermodynamic effects at elevated temperatures in a small Venturi channel. Int. J. Heat Mass Transfer.

[6] Ge M., Zhang G., Petkovšek M., Long K. (2022). Intensity and regimes changing of hydrodynamic cavitation considering temperature effects. J. Cleaner Prod..

[7] Jones I.R., Edwards D.H. (1960). An experimental study of the forces generated by the collapse of transient cavities in water. J. Fluid Mech..

[8] Liu W., Kang Y., Zhang M., Wang X., Li D. (2017). Self-sustained oscillation and cavitation characteristics of a jet in a Helmholtz resonator. Int. J. Heat Fluid Flow.

[9] Fang Z., Zeng F., Xiong T., Wei W., Jiang P., Wu Q., Wang Y., Fei Y. (2020). Large eddy simulation of self-excited oscillation inside Helmholtz oscillator. Int. J. Multiphase Flow.

[10] Fan C., Zhang M., Liu Q., Zhang X., Kang Y., Li D. (2024). High-speed observation of the flow characteristics of self-excited oscillation pulsed abrasive SC-CO2 jet. Fuel.

[11] Liu Y., Wei J., Ren T. (2016). Analysis of the stress wave effect during rock breakage by pulsating jets. Rock Mech. Rock Eng..

[12] Chen Y., Fang Z., Xiong T., Hou W., Zhang Z., Shi R. (2023). An experimental study on the erosion of sandstone by self-excited oscillation cavitation waterjet in submerged environment. Ocean Eng..

[13] Huang M., Kang Y., Wang X., Hu Y., Cai C., Liu Y., Chen H. (2018). Experimental investigation on the rock erosion characteristics of a self-excited oscillation pulsed supercritical CO2 jet. Appl. Therm. Eng..

[14] Foldyna J., Sitek L., Švehla B., Švehla Š. (2004). Utilization of ultrasound to enhance high-speed water jet effects. Ultrason. Sonochem..

[15] Li D., Kang Y., Wang X., Ding X., Fang Z. (2016). Effects of nozzle inner surface roughness on the cavitation erosion characteristics of high speed submerged jets. Exp. Therm Fluid Sci..

[16] Cai T., Liu B., Ma F., Pan Y. (2020). Influence of nozzle lip geometry on the Strouhal number of self-excited waterjet. Exp. Therm Fluid Sci..

[17] Cai T., Pan Y., Ma F. (2020). Effects of nozzle lip geometry on the cavitation erosion characteristics of self-excited cavitating waterjet. Exp. Therm Fluid Sci..

[18] H. Shi, Y. Kang, D. Li, Z. Fang, Effects of the exit aspect ratio of organ-pipe nozzle on the axial pressure oscillation characteristics of self-resonating waterjet, Proc. Inst. Mech. Eng. Part C J. Mech. Eng. Sci. 234.23 (2020): 4589-4598, https://doi.org/10.1177/0954406220925837.

[19] Xiang L., Wei X., Chen S. (2020). Experimental study on the frequency characteristics of self-excited pulsed cavitation jet. Eur. J. Mech. B. Fluids.

[20] Liu X., Xu H., Zhao L., Yu X., Chen H., Zhang S., Ji J. (2021). Investigation of the impact characteristics and pulse mechanism of a self-excited aspiration pulsed jet device. Exp. Therm Fluid Sci..

[21] Zhang J., Zhang B., Liu B., Li B. (2024). Investigation on the influence of the frequency of pulsed water jet on the rock-breaking effect. Powder Technol..

[22] Wang D., Wang Z., Fan Y., Sun X., Gao Q. (2023). Characterization of vortex structures with self-excited oscillations based on Liutex-Omega vortex identification method. J. Hydrodyn..

[23] Fang Z., Hou W., Fan S., Guo X., Chen Y. (2024). Coherent structure analysis of cavitation waterjets using dynamic mode decomposition. Phys. Fluids.

[24] Zdanowski F., Malico I. (2025). CFD analysis of twin turbulent plane jets confined by walls: Effects of geometry on flow dynamics and heat transfer. Appl. Therm. Eng..

[25] Wu Q., Wei W., Deng B., Jiang P., Li D., Zhang M., Fang Z. (2019). Dynamic characteristics of the cavitation clouds of submerged Helmholtz self-sustained oscillation jets from high-speed photography. J. Mech. Sci. Technol..

[26] Hou B., Hou W., Gao Q., Wang Y., Guo X., Fang Z. (2025). The kinetic mechanism of vortex-cavitation interaction in dual-chamber self-excited oscillation waterjets. Phys. Fluids.

[27] Wang J., Wang Z., Xu Y., Yan Y., Xu X., Li S. (2024). Evolution of cavitation clouds under cavitation impinging jets based on three-view high-speed visualization. Geoenergy Sci. Eng..

[28] Peng C., Tian S., Li G. (2018). Joint experiments of cavitation jet: High-speed visualization and erosion test. Ocean Eng..

[29] Xu S., Long X., Wang J., Cheng H., Zhang Z. (2022). Experiment on flow dynamics and cavitation structure in an axisymmetric venturi tube based on xt diagrams and proper orthogonal decomposition. Exp. Therm Fluid Sci..

[30] Hu J., Yuan M., Feng G. (2023). Experimental investigation on the cavitation modulation mechanism in submerged self-sustained oscillating jets[J]. Ocean Eng..

[31] Fang Z., Hou W., Xu Z., Guo X., Zhang Z., Shi R., Yao Y., Chen Y. (2023). Large eddy simulation of cavitation jets from an organ-pipe nozzle: the influence of cavitation on the vortex coherent structure. Processes.

[32] Wang L., Wu W., Li X., Fan S., Fang Z. (2025). Coupling mechanism of structure–cavitation impact in reflux self-excited oscillating nozzles. Ocean Eng..

[33] Ge M., Manikkam P., Ghossein J., Subramanian R.K., Coutier-Delgosha O., Zhang G. (2022). Dynamic mode decomposition to classify cavitating flow regimes induced by thermodynamic effects. Energy.

[34] Gong J., Luo W., Wu T., Zhang Z. (2022). Numerical analysis of vortex and cavitation dynamics of an axial-flow pump. ENG APPL COMP FLUID..

[35] Ye Q., Wang Y., Shao X. (2023). Dynamics of cavitating tip vortex. J. Fluid Mech..

[36] Ausoni P., Farhat M., Escaler X., Egusquiza E., Avellan F. (2007). Cavitation influence on von Kármán vortex shedding and induced hydrofoil vibrations. J. Fluids Eng..

[37] Fang Z., Ji Z., Kang D., Chen Y., Zhang X., Wang S., Xiong T. (2023). Cavitation damage characteristics following marine fouling cleaning by a self-excited oscillation cavitation waterjet. Appl. Ocean Res..

[38] Wang P., Li Z., Ni H., Liu Y., Dou P. (2020). Experimental study of rock breakage of an interrupted pulsed waterjet. Energy Rep..

[39] Pan Y., Ma F., Liu B., Cai T. (2020). Cavitation intensity and erosion pattern of a self-excited cavitating jet. J. Mater. Process. Technol..

[40] Kerboua K., Hamdaoui O. (2018). Ultrasonic waveform upshot on mass variation within single cavitation bubble: Investigation of physical and chemical transformations. Ultrason. Sonochem..

[41] Ma X., Huang B., Wang G., Zhang M. (2017). Experimental investigation of conical bubble structure and acoustic flow structure in ultrasonic field. Ultrason. Sonochem..

[42] Lv Z., Hou R., Tian Y., Huang C., Zhu H. (2018). Investigation on flow field of ultrasonic-assisted abrasive waterjet using CFD with discrete phase model. Int. J. Adv. Manuf. Technol..

[43] Lv Z., Hou R., Tian Y., Huang C., Zhu H. (2018). Investigation on flow field of ultrasonic-assisted abrasive waterjet using CFD with discrete phase model. Int. J. Adv. Manuf. Technol..

[44] Lv Z., Hou R., Wang T., Huang C., Zhu H. (2019). Research on cavitation involved in ultrasonic-assisted abrasive waterjet machining. Int. J. Adv. Manuf. Technol..

[45] Qin D., Lei S., Zhang B., Liu Y., Tian J., Ji X., Yang H. (2024). Influence of interactions between bubbles on physico-chemical effects of acoustic cavitation. Ultrason. Sonochem..

[46] Coulombel P., Denner F. (2024). Modeling time-delayed acoustic interactions of cavitation bubbles and bubble clusters. Phys. Fluids.

[47] Huang T., Zhang J., Ye J., Gao Z. (2025). Numerical Study on the Dynamics and thermal Effects of Bubble Stable Cavitation in Focused Ultrasound Fields. Processes.

